# Strength of Religious Faith and Attitude Towards Euthanasia Among Medical Professionals and Opinion Makers

**DOI:** 10.1007/s10943-023-01860-1

**Published:** 2023-07-18

**Authors:** Andrzej Guzowski, Joanna Fiłon, Elżbieta Krajewska-Kułak

**Affiliations:** Department of Integrated Medical Care, Faculty of Health Sciences, Medical University of Bialystok, Ul. Marii Sklodowskiej-Curie 7A, 15-096 Bialystok, Poland

**Keywords:** Euthanasia, Futile medical care, Religious faith

## Abstract

The aim of the research was to examine whether the strength of religious faith among health professionals, politicians, journalists and religious leaders in Poland influences their knowledge and attitudes towards the withdrawal of futile care, and euthanasia. The study was carried out using a group of 449 respondents employed in medical professions (nurses, midwives and paramedics), and 142 respondents of non-medical professions (politicians, journalists and clergymen). The method used was a diagnostic survey with an original, anonymous Internet survey, as well as the standardised Santa Clara Strength of Religious Faith Questionnaire (SCSORF). It has been demonstrated that the greater the influence of religion on a person’s life, the lesser their tolerance for the refusal of life-saving/life support procedures.

## Introduction

Discussing euthanasia is a very delicate subject because it concerns the most intimate issue in a person’s life, i.e. the autonomous decision to continue or end life. However, such an approach to this problem is an oversimplification, and therefore, possible definitions of euthanasia should be given. Nowadays, there is no debate on euthanasia in Poland, and this fact needs to be emphasised. In his research, Abohaimed et al. (2019) distinguishes four types of euthanasia: active, passive and indirect euthanasia as well as assisted suicide. In the discussion on this article, Varkey (2020) points to the need to clarify the meaning of the term euthanasia in various variants provided by Abohaimed et al. Varkey indicates the differences in understanding the term indirect euthanasia and stresses the importance of patient autonomy in the process of decision-making. In the author’s opinion, passive and indirect modes are not considered euthanasia: “Withdrawal or discontinuation of life-support treatment at the patient’s request is not euthanasia as it is based on ‘patient autonomy’, one of the fundamental principles of medical ethics” (Varkey, 2020). Another point of understanding, especially of passive euthanasia, is proposed by the Team of Experts of the Polish Episcopal Conference for Bioethics. “If, in the face of the irreversible progressive disappearance of life processes, the doctor has done his/her best, then the decision to discontinue further therapy, i.e. to resign from using measures disproportionate to the situation, cannot be considered immoral by neither the doctor nor other people; it would certainly be a futile therapy, the interruption of which cannot be qualified as euthanasia” (Team of Experts of the Polish Episcopal Conference for Bioethics, 2018). So what is the difference between cessation of futile medical care and passive euthanasia? The main element differentiating the withdrawal from persistent therapy from passive euthanasia is the intention of the doctor. The intention is the initial and constant motive of the physician’s conscious action for a specific purpose. There is a fundamental difference between “killing” and “accepting the process of dying”. In the first case, this is murdering a person, while in the second, it is accepting the process with all its consequences (The Pontifical Council for Pastoral Assistance to Health Care Workers, 1995). It is the discontinuation of persistent/futile therapy recommended by the Polish Paediatric Society in the guidelines for physicians on withdrawal from persistent life-support treatment in children and in the guidelines of the Polish Society of Anaesthesiology (Kübler et al., 2014). Both documents mention the cessation of futile medical care and not the use of passive euthanasia.

A difficulty in distinguishing passive euthanasia from futile medical care results from the use of the unclear term of intention. Another issue is the lack of legal regulations in Poland regarding consent to euthanasia and the methods of carrying out this procedure. There is also no definition of euthanasia so far—in other words, there is no such thing as euthanasia in the Polish law. There is merely a mention of euthanasia in the Penal Code (Article 150 of the Penal Code), which qualifies euthanasia as a special, privileged type of homicide with the mentioning of corresponding criminal liability (Act of 6 June 1997 promulgating the Penal Code, 1997). However, this one sentence is too general to define euthanasia in Polish law. Finally, the third and probably the most important issue that may explain some of our research results is the fact that there is no honest, genuine discussion on euthanasia in Poland—the problem seems to be overlooked.

Szkocik (2016) suggested that religious convictions and religion in general tend to be construed in terms of their social utility, as providing a motivation for cooperation and being perceived through the prism of their alleged truthfulness. According to the author (Szkocik, 2016), a religious person believing in certain dogmatic propositions usually either considers those to be true, or does not subject them to analysis. On the one hand, such persons accept the validity of the beliefs they consider to be true and to provide an appropriate motivational power. On the other hand, however, they are prone to the phenomena of “theological incorrectness” and half-hearted beliefs, associated with the situations when believers suspend or abandon their religious convictions (Szkocik, 2016).

In the relevant literature, it has been indicated that more religious patients tend to cope better when faced with illness, suffering, and disability (Idler & Kasl, 1997; Matthews et al., 1998; Walczak et al., 2018). However, at times, strongly religious persons have unrealistic demands and expectations of themselves, and that may lead to isolation, stress, anxiety or alienation within groups that do not share their beliefs (Mueller et al., 2001).

The American Psychiatric Association recommends that doctors ask their patients about their religious orientation and spiritual condition in order to better tailor the treatment to the sick person. Furthermore, a special questionnaire was developed to evaluate patients’ religious attitude (HOPE) (Anandarajah & Hight, 2001), as well as an algorithm for the conduct during an interview on the subject (FICA) (Puchalski & Romer, 2000). Mueller et al. (2001) expressed their conviction that clinicians ought to know the spiritual condition and the religious needs of their patients and should attempt to meet those needs for several reasons; most patients consider their physical and spiritual health to be of equal importance; religious faith helps patients cope with disease; an understanding of the religious and spiritual needs of patients fosters the doctor–patient relationship.

The aim of the study was to examine whether the strength of religious faith among health professionals, politicians, journalists and religious leaders in Poland influences their knowledge and attitude towards the withdrawal of futile care and euthanasia.

## Materials and Methods

The analysis covered a sample of 449 respondents representing medical professions (104 doctors, 273 nurses, 27 midwives and 45 paramedics) in group I and a sample of 142 persons representing non-medical professions (19 politicians, 42 journalists and 81 members of the clergy) in group II from all over Poland.

The study was performed using the method of diagnostic survey with an original, anonymous Internet survey, and a standardised Santa Clara Strength of Religious Faith Questionnaire (SCSORF).

An original diagnostic survey was used to collect sociodemographic data. The survey included questions about gender, age, place of residence, marital status, education, associations with the concept of euthanasia and the cessation of futile medical care, as well as opinions on euthanasia: Does euthanasia mean the cessation of futile therapy and life-saving procedures in end-stage disease? Is the cessation of futile medical care the same as the cessation of treatment? Who should make the decision to cease futile therapy? Does the patient have the right to cease futile medical care against the doctor’s will and to object to life-saving medical procedures?

The Santa Clara Strength of Religious Faith Questionnaire (SCSORF) consists of ten questions regarding religious beliefs, independent of the religion professed by the respondents. The higher the score obtained in the questionnaire, the greater the degree of religiosity. Depending on the researched population, Cronbach’s alpha varies between 0.93 and 0.97. The Polish version of the questionnaire was used with permission from Dr. M. Wnuk (Freiheit et al., 2006; Lewis et al., 2005; Plante & Boccaccini, 1997a, 1997b; Sherman et al., 1999, 2001; Strawser et al., 2004; Wnuk, 2009). Respondents could choose not to answer a question without providing a reason at any stage.

The survey was posted on closed internet forums for doctors, nurses, midwives and paramedics, and sent by e-mail by organizations associating medical professionals (e.g. medical and nursing chambers).

Clergy—the survey was sent to the curia and religious orders all over Poland with a request to make it available to clergymen.

Journalists—the survey was sent to the *Polish Journalists Association* with a request to make it available to the members.

Politicians—the survey was sent by e-mail to the members of the Parliament.

### Statistical Analysis

The statistical analysis of the data was performed using the using StatSoft, Inc. (2017) software STATISTICA (data analysis software system), version 13.3. Statistical analysis of the results included calculating mean values, standard deviation, median, and the minimum and the maximum of the strength of religious faith in the studied groups.

In order to perform further analysis, the researched party was split into three groups based on the strength of their religious faith. The division was based on the value of the lower and upper quartiles of the measure of the strength of religious faith, expressed in points, thus identifying a group of persons displaying a low intensity of religious convictions, a group with average intensity of religious convictions, and a group of highly religious persons. The evaluation of the differences in the distribution of answers to the respective questions with regard to religious faith was performed using the Chi-square independence test. The statistical significance level was accepted as *p* ≤ 0.05.

## Results

Table 1 shows the basic social and demographic characteristics of the two compared groups.

**Table 1:** Basic social and demographic characteristics of the studied groups

Gender	Profession	
	Medical	Non-medical
*Gender of the researched persons*		
Male	18.8%	75.2%
Female	81.2%	24.8%
*Level of education*		
Secondary education	10.0%	13.1%
Higher education	90.0%	86.9%
*Age*		
20–30 Years of age	34.5%	22.1%
30–40 Years of age	19.0%	33.1%
40–50 Years of age	16.2%	13.1%
50–60 Years of age	30.3%	31.7%
*Place of residence*		
Rural area	15.3%	17.5%
Small city	32.3%	23.2%
Large city	52.4%	59.3%

As demonstrated in the tallies below, the groups were compared with regard to their age structure, education level and age. However, they differed rather noticeably in their gender compositions—with the group of medical professionals being dominated by females, whereas the non-medical group by males. The difference in the gender composition is consistent with health-care professions being dominated by a female workforce, particularly in the positions of nurses and midwives. Meanwhile, the non-medical professions considered in the study are male dominated—entirely in the case of clerics, and to a large extent in the case of politicians. The differences in views on euthanasia and futile medical care may therefore result from differences in the gender structure and not from the fact of not belonging to a certain professional group.

In line with the expectations, the evaluation of the strength of religious faith using the standardised SCSORF questionnaire demonstrated that clergymen stood out among the researched groups, as they were reported to have the greatest strength of religious convictions. In the case of the remaining groups, the level of faith did not vary as much, though it was indeed much higher, say, among nurses than among paramedics (on average: 34.2 pts vs. 26.9 pts). The results are presented in Table 2.

**Table 2:** Strength of religious faith in the studied group

Profession	Strength of religious faith					
	*N*^1)^	$\overline{x}$	Me	*s*	min	max
Doctors	104	28.6	28	14.3	10	50
Nurse	273	34.2	36	13.0	10	50
Midwife	27	28.0	29.5	14.0	10	50
Paramedic	45	26.9	31	13.4	10	50
Politician	19	34.7	41	14.7	10	50
Journalist	42	30.0	32	13.1	10	50
Clergyman	81	48.3	50	4.2	23	50

^1)^The counts are given in the table as not all subjects completed the SCSORF questionnaire

Regardless of the role of religion in their lives, the majority of medical workers considered the withdrawal of futile treatment to be equivalent to euthanasia of the patient. The responses regarding the evaluation of euthanasia proved similarly structured. People with strong religious faith were definitely the least inclined to consider euthanasia an appropriate solution even for the terminally ill. It was solely the suggestion of euthanasia as a means to avoid expensive treatment that was entirely rejected by a great majority of respondents, regardless of their religious convictions. The religious positions among the group of medical professionals determined their attitude towards the withdrawal of futile treatment. It turned out that the lower the importance of religion in the lives of the surveyed persons, the greater their acceptance of the withdrawal of futile medical care. No influence of religiosity was found on the recognition of the withdrawal of futile treatment as equivalent to the termination of treatment. In the latter case, the majority of medics in all groups disagreed with the statement. The results are presented in Table 3.

**Table 3:** Perception of the withdrawal of futile treatment as euthanasia and associations with these notions

	Strength of religious faith			*p*
	Low	Average	High	
*Is the withholding of futile medical care equivalent to euthanasia?*				
Health-care professionals				
Yes	91.8%	94.7%	90.8%	*p* = 0.4011
No	8.2%	5.3%	9.2%	
Non-health-care professionals				
Yes	87.5%	76.9%	75.5%	*p* = 0.3842
No	12.5%	23.1%	24.5%	
*Is the withdrawal of futile medical care equivalent to the termination of treatment?*				
Health-care professionals				
Yes	4.1%	6.2%	11.7%	*p* = 0.1922
No	94.8%	90.7%	86.4%	
I don’t know	1.0%	3.1%	1.9%	
Non-health-care professionals				
Yes	12.1%	23.1%	14.5%	*p* = 0.1452
No	69.7%	71.2%	80.0%	
I don’t know	18.2%	5.8%	5.5%	
*Associations with the notion of euthanasia*				
Health-care professionals				
Relief from suffering	76.3%	45.1%	29.1%	0.0000***
Protection of the patient’s dignity	44.3%	24.4%	4.9%	0.0000***
Providing a dignified death	50.5%	24.9%	6.8%	0.0000***
Expensive treatment	9.3%	6.7%	5.8%	0.6105
Infliction of death	44.3%	58.5%	81.6%	0.0000***
Non-health-care professionals				
Relief from suffering	54.5%	19.2%	14.5%	0.0001***
Protection of the patient's dignity	33.5%	7.7%	1.8%	0.0000***
Providing a dignified death	33.3%	11.5%	1.8%	0.0001***
Expensive treatment	12.1%	19.2%	20.0%	0.6129
Infliction of death	48.5%	88.5%	96.4%	0.0000***
*Association with the notion of withdrawal of futile care*				
Health-care professionals				
Relief from suffering	78.4%	63.7%	55.3%	0.0025**
Protection of the patient’s dignity	78.4%	64.8%	62.1%	0.0273*
Providing a dignified death	77.3%	70.5%	68.0%	0.3094
Expensive treatment	25.8%	22.8%	10.7%	0.0149*
Infliction of death	0.0%	2.1%	7.8%	0.0033**
Non-health-care professionals				
Relief from suffering	57.6%	40.4%	25.5%	0.0106*
Protection of the patient’s dignity	48.5%	44.2%	54.5%	0.5630
Providing a dignified death	51.1%	36.5%	45.5%	0.5630
Expensive treatment	15.2%	23.1%	10.9%	0.2307
Infliction of death	9.1%	26.9%	25.5%	0.1165

Chi-square independence test was used;

^*^–*p* < 0.05 (statistically significant)

^**^–*p* < 0.01 (highly statistically significant)

^***^–*p* < 0.001 (very highly statistically significant)

A great majority of respondents from the group of non-medical professionals (between ca. 75% and ca. 87%), regardless of their religious faith, believed that the withdrawal of futile care is equivalent to euthanasia of a patient; however, in that regard, there was no statistically significant correlation between the strength of religious convictions and the distribution of opinions. Only to a small extent did religious convictions in that group influence the associations with the notion of futile care withdrawal. The single statistically significant difference was that in the percentage of persons considering the withdrawal of futile treatment to constitute the alleviation of suffering—the number of such persons was relatively higher when the strength of religious faith was low, whereas it dipped with the increase in the strength of religious faith. The persons from the non-medical professionals group with strong religious beliefs were by far the least inclined to ever consider euthanasia an appropriate solution (alleviation of suffering, providing a dignified death, dignity of the patient) even for the terminally ill. Only the acceptance of euthanasia as a means of avoiding expensive treatment was decisively rejected by a great majority of respondents regardless of their religious beliefs, and there were no statistically significant differences between the compared non-medical professions. Religiosity did not bear on non-medics’ assessment of the withdrawal of futile care as the termination of treatment. Regarding that issue, a majority of respondents in either group (ca. 70–80%) did not agree with the statement. The results are presented in Table 3.

Regardless of the strength of their religious faith, medical professionals were of the opinion that the decision to withdraw futile treatment ought to be taken by the patient together with the clinician. The percentage of respondents acknowledging the patient’s right to terminate futile treatment even against the doctor’s recommendation in this group was the highest among all the surveyed persons who defined their religious involvement as low. In that case, the correlation was statistically significant. The greater the influence of religion on the lives of medics, the lower their tolerance of the patient’s right to refuse life-saving procedures. Among the respondents seeing their religious convictions as the weakest, the percentage of persons in favour of the right was 100%. The results are provided in Table 4.

**Table 4:** Strength of religious faith and the decision to terminate futile care

Strength of religious faith			
	Low	Average	High
*Who should decide about the withdrawal of futile care?*			
Health-care professionals	*p* = 0.5267		
Patient	17.1%	19.1%	15.2%
Clinician	10.2%	4.6%	6.5%
Patient’s family	3.4%	6.9%	4.4%
Patient and clinician	69.3%	69.4%	73.9%
Non-health-care professionals	*p* = 0.2709		
Patient	36.7%	26.1%	26.5%
Clinician	3.3%	10.9%	10.2%
Patient’s family	0.0%	2.2%	10.2%
Patient and clinician	60.0%	60.9%	53.1%
*Patient’s right to terminate futile care against clinician*'*s recommendation*			
Health-care professionals	*p* = 0.0070**		
Yes	93.8%	77.7%	75.7%
No	2.1%	12.5%	13.6%
I don’t know	4.1%	9.8%	10.7%
Non-health-care professionals	*p* = 0.0001***		
Yes	81.8%	51.9%	36.4%
No	3.0%	34.6%	54.5%
I don’t know	15.2%	13.5%	9.1%
*Patient’s right to refuse life-saving medical procedures*			
Health-care professionals	*p* = 0.0026**		
Yes	100.00%	93.3%	85.4%
No	0.00%	3.1%	7.8%
I don’t know	0.00%	3.6%	6.8%
Non-health-care professionals	*p* = 0.0000***		
Yes	93.9%	59.6%	22 (40.0%)
No	3.0%	21.2%	26 (47.3%)
I don’t know	3.0%	19.2%	7 (12.7%)

Chi-square independence test was used

^*^–*p* < 0.05 (statistically significant)

^**^–*p* < 0.01 (highly statistically significant)

^***^–*p* < 0.001 (very highly statistically significant)

The respondents employed in non-medical professions, regardless of the strength of their religious faith, were more often than not of the opinion that it is the patient together with the clinician who should take the decision to withdraw futile care. No statistically significant correlation was found between this issue and the strength of religious faith (the *p*-value much above 0.05). The percentage of persons in favour of the patient’s right to terminate futile treatment even contrary to the doctor’s recommendation proved highest among the respondents defining their religious involvement as low—and gradually decreased with the increase in the strength of religious faith. The correlation was statistically highly significant (the *p*-value being *p* = 0.0001***). The greater the influence of religion on the lives of respondents, the lower their tolerance of the patient’s right to refuse life-saving procedures. Among the surveyed persons who assessed their religious faith as the weakest, the percentage of people in favour of such a right reached almost 100%. Of course, such pronounced differences with a relatively high number of respondents in the compared groups are highly significant statistically (*p* = 0.0000***). The results are provided in Table 4.

In the group of medical professionals, in the case of three life-saving procedures, that is, the withdrawal of dialysis, the refusal of blood transfusions, and the withholding of antibiotic treatment, the strength of religious faith proved to be of significance. Persons who declared that they consider faith important were more often of the opinion that the withdrawal of the above kinds of treatment is equivalent to euthanasia. The situation was different in the case of perceiving the withdrawal of certain treatments as the termination of futile care. With regard to that greater religiosity tended to weaken the evaluation of the failure to perform certain procedures as the withdrawal of futile care (Table 5).

**Table 5:** Strength of faith and the opinion whether the withdrawal/withholding of life-saving procedures at the terminal stage of a disease is euthanasia/termination of futile care

	Strength of religious faith			
	Low	Average	High	*p*
*Is the withdrawal of life-saving procedures from patients in the terminal stage of a disease equivalent to euthanasia?^*				
Health-care professionals				
Withholding of ventilator treatment	2.1%	8.3%	6.8%	0.1195
Withholding of antibiotic treatment	5.2%	12.4%	13.6%	0.1045
Withholding of parenteral nutrition	22.7%	33.7%	35.0%	0.1055
Abandonment of surgical procedures	1.0%	4.7%	6.8%	0.1277
Withholding of dialysis	5.2%	15.5%	12.6%	0.0384*
Withholding of blood transfusions	5.2%	16.6%	10.7%	0.0169*
Withdrawal of antibiotic treatment	4.1%	17.6%	21.4%	0.0014**
Withdrawal of parenteral nutrition	22.7%	33.7%	36.9%	0.0719
Withdrawal of dialysis	12.4%	21.2%	23.3%	0.1084
Non-health-care professionals				
Withholding of ventilator treatment	21.2%	25.0%	45.4%	0.0236*
Withholding of antibiotic treatment	15.2%	28.8%	34.5%	0.1423
Withholding of parenteral nutrition	27.3%	50.0%	52.7%	0.0499*
Abandonment of surgical procedures	15.2%	25.0%	25.5%	0.4826
Withholding of dialysis	21.2%	34.6%	40.0%	0.1916
Withholding of blood transfusions	15.2%	32.7%	40.0%	0.0505
Withdrawal of antibiotic treatment	18.2%	36.5%	41.8%	0.0706
Withdrawal of parenteral nutrition	30.3%	44.2%	58.2%	0.0368*
Withdrawal of dialysis	27.3%	36.5%	50.9%	0.0738
*Is a failure to attempt these life-saving procedures equivalent to withdrawal of futile medical care?^*				
Health-care professionals				
Withholding of ventilator treatment	89.7%	80.8%	72.8%	0.0100**
Withholding of antibiotic treatment	58.8%	62.7%	56.3%	0.5407
Withholding of parenteral nutrition	46.4%	44.6%	43.7%	0.9253
Abandonment of surgical procedures	85.6%	85.0%	70.9%	0.0058**
Withholding of dialysis	77.3%	75.1%	67.0%	0.1986
Withholding of blood transfusions	83.5%	73.6%	68.0%	0.0377*
Withdrawal of antibiotic treatment	71.1%	66.3%	52.4%	0.0138*
Withdrawal of parenteral nutrition	51.5%	48.2%	41.7%	0.3613
Withdrawal of dialysis	75.3%	63.7%	57.3%	0.0259*
Non-health-care professionals				
Withholding of ventilator treatment	60.6%	50.0%	32.7%	0.0291*
Withholding of antibiotic treatment	72.7%	44.2%	40.0%	0.0079**
Withholding of parenteral nutrition	27.3%	50.0%	52.7%	0.0499*
Abandonment of surgical procedures	75.8%	50.0%	45.5%	0.0162*
Withholding of dialysis	63.6%	40.4%	32.7%	0.0161*
Withholding of blood transfusions	72.7%	44.2%	30.9%	0.0007***
Withdrawal of antibiotic treatment	69.7%	36.5%	30.9%	0.0010**
Withdrawal of parenteral nutrition	30.3%	44.2%	58.2%	0.0368*
Withdrawal of dialysis	54.5%	44.2%	23.6%	0.0089**

Chi-square independence test was used;

^*^–*p* < 0.05 (statistically significant)

^**^–*p* < 0.01 (highly statistically significant)

^***^–*p* < 0.001 (very highly statistically significant)

^The values do not add up to 100% as multiple answers were allowed

To summarise the results obtained, it is worth pointing out the fact that even in the group of medical professionals, the issues of outlook have a statistically significant influence on attitudes towards euthanasia and futile medical care.

The differences in the distribution of answers were not as great as in the case of non-medical processionals; nevertheless, it turned out that faith, conscience, and religiosity may influence attitudes to performing certain medical procedures.

## Discussion

Koenig et al. (2004) identify five types of spirituality: the humanistic stance (directed at the self-transcending human spirit, unrelated to any religion, with a highly developed ethical system), an undefined attitude (cultural elites, individualism, with no affiliation to any institution, demonstrating beliefs in energy, nature, astrology, para-psychology), Eastern religiosity (Buddhism, Hinduism, Taoism, Shinto, and others), type I of Western spirituality (to be found in most Christian, Muslim, and Jewish denominations; among its characteristic features there is the sense of dependence on a personal God, an intimate relationship with God taking the form of specific prayer, the striving to determine God’s will, and the belief in divine interventions in the world) and type II of Western spirituality (most Christians, Muslims, and Jews in the USA; belief in the dependence of human life on God, but a less direct relationship with him, less specific, more reflective prayers, weaker striving to understand God’s will).

In the authors’own research, the standardised Santa Clara Strength of Religious Faith Questionnaire (SCSORF) was used, as it enables the measurement of the strength of religious faith regardless of the actual religious persuasion and religious practice typical for a given religious dogma. We were able to find that that the level of faith, with the exception of clergymen (displaying a decisively greatest strength of religious convictions), was not as varied in other groups, but—for instance—it was higher among nurses than among paramedics, midwives, and clinicians.

Regardless of the measurements of religiosity used, the results of research performed by many authors (Bachman et al., 1996; DeCesare, 2000; Hamil-Luker & Smith, 1998; Holden, 1993; MacDonald, 1998; Seale & Addington-Hall, 1994) unequivocally point to the key role played by religious attitudes in the evaluation of euthanasia. Regardless of their confession and of the measurement of religiosity used, religious persons are against euthanasia, particularly as regards the active form thereof. A study performed in 2004 by the Gallup Organisation among citizens of the USA demonstrated that the frequency of church attendance is correlated with a negative attitude towards euthanasia (as cited in: Wnuk, 2015). In a study by Givens and Mitchell (2009) performed on a group of 786 Americans, it was demonstrated that 70.6% of them were in favour of euthanasia in the case of a terminal illness. Those more religious among them more often declared their objections to euthanasia (Givens & Mitchell, 2009). Among adult Australians, Ho and Penney (1992) found that the more religious they are, the more conservative their outlook; thus, they take a stronger stance against passive and active euthanasia.

In this study, regardless of the role of religion in their lives, a great majority of medical professionals and most of the group of non-medical professionals considered the withdrawal of futile treatment to be equivalent to euthanasia of the patient. Meanwhile, the persons with greater strength of religious beliefs were decidedly the least likely to regard euthanasia as an appropriate solution even for the terminally ill. The lower the importance of religion in the lives of the surveyed medics, the greater their acceptance of the withdrawal of futile care. Persons from the group of non-medical professions with greater strength of religious faith were markedly the least inclined to consider euthanasia an appropriate solution (alleviation of suffering, providing a dignified death and dignity of the patient) even for the terminally ill. Only the suggestion of euthanasia as a means to avoid expensive treatment was absolutely rejected by a great majority of respondents, regardless of their religious attitudes. No statistically significant differences between the compared non-medical professions were recorded. Religious beliefs did not influence non-medics’ evaluation of the termination of futile care as the termination of treatment.

Musgrave and Soudry (2000), studying the relationship between religiosity and euthanasia in a group of nurses, were able to ascertain that religiousness, understood as belonging to a religious confession, involvement in religious practices, and the importance of religion treated as an outlook, constitutes an important correlate for attitudes towards euthanasia. The midwives who were more religious proved to be opponents of active euthanasia. Those who practiced in countries where the law is more liberal towards euthanasia were more in favour of that solution (Musgrave & Soudry, 2000). Meanwhile, in a study performed among religious Japanese persons of various confessions, religiosity turned out to be connected to attitudes towards euthanasia. From among all of the researched persons, the least in favour of euthanasia were Catholics (Tanida, 2000). Furthermore, religious clinicians in Mexico were reported to be greater opponents to euthanasia than their non-religious counterparts (Lisker et al., 2008).

In this study, the majority of both medical and non-medical professionals, regardless of the strength of their religious faith, were of the opinion that the decision to withdraw futile care should be taken by the patient together with the clinician. The percentage of persons in favour of the patient’s right to terminate futile care even against the doctor’s recommendation proved greatest in the group of respondents describing their religious involvement as weak. Meanwhile, in the group of non-medics, the percentage of persons supporting the patient’s right to terminate futile treatment, even against the doctor’s recommendation, was the highest among the respondents declaring their religious involvement to be weak. Among the respondents evaluating their own religiosity as the lowest, the percentage of persons in favour of such a right reached almost 100%.

A study on a group of Israeli septuagenarians and above demonstrated that religiosity was directly and indirectly associated with the willingness to accept life support measures, whereas the indirect variables with regard to it included the will to live, the fear of death, and the fear of dying (Carmel & Mutran, 1997).

In the authors’ own research, medical professionals who saw faith as important were statistically significantly more often of the opinion that the withdrawal of dialysis, the termination of blood transfusions, and the withholding of antibiotics does in fact amount to euthanasia. In the non-medical group, greater religiosity tended to weaken the evaluation of failure to perform certain procedures as a termination of futile therapy.

In an age of progress in medical sciences and technologies, man has greater control over the world, including over the process of dying (Tokarz, 2012). Contemporary medicine uses its abilities right up until the moment when any activity proves obviously futile or harmful. The above leads to the blurring of the boundary not only between life and death, but also between human activity and natural processes. On numerous occasions, futile care proved able to save human life, while concurrently enabling the clinician to demonstrate their courage and mastery of medical practice. Regretfully, it also provided patients with false hope, for aggressive therapeutic procedures leading to the extension of the terminal stage of the disease, causing additional suffering (Tokarz, 2012).

### Limitations

In this study, there are several limitations that could be solved in future studies. The main curtailment of our work is the small number of cases (especially a sample of persons representing non-medical professions) and strong regional focus of this study. The relatively narrow group of only 27 midwives and 45 paramedics is not representative of these medical professions. Our sample size is small, though fully statistically powered for the type of analysis used.

Our method of recruitment may have resulted in a small amount of bias as the samples were self-selecting; however, we have made considerable effort to reduce this possibility and ensure that participants can be completely honest in their responses through careful anonymity procedures.

Another issue in the Polish law is the lack of definition of euthanasia. There is no legal regulations in Poland regarding consent to euthanasia and the methods of carrying out this procedure. There are also a difficulty in distinguishing passive euthanasia from futile medical and understand of what constitutes euthanasia is problematic for respondents, especially those who have a strong religious faith.

Final, but probably, the most important issue that may explain some of our research results is the fact that there is no honest, genuine discussion on euthanasia in Poland—the problem seems to be overlooked.

This study is relatively small and should be the beginning of a larger exploration of views in this area. As part of future research, it would be useful to examine the influence of geographical location (different countries) has on attitudes towards Attitude Towards Euthanasia. Finally, is there a disparity between beliefs between an urban and rural within countries?

## Conclusions

1. An outlook on life (faith, conscience and religiosity) has a significant statistical impact on the approach to certain life-saving medical procedures and on the attitude towards euthanasia and futile medical care.
2. In the groups of medical and non-medical professionals, the persons with greater strength of religious faith were the least inclined to consider euthanasia an appropriate solution even for the terminally ill.
3. In the group of medical professionals, the lower the importance of religion in the lives of the respondents, the greater their acceptance of the withdrawal of futile care; meanwhile, in the group of non-medical professionals, their religiosity did not have such influence.
4. Regardless of the strength of their religious faith, the respondents were of the opinion that the decision to terminate futile treatment should be taken by the patient together with the clinician.
5. The greater the influence of religion on the lives of the persons from both researched groups, the lower their tolerance of the refusal of life saving/support procedures.
6. In the group of medical professionals, the persons who regarded faith as something important were more often persuaded that the termination of life-saving procedures, that is, the withdrawal of dialysis, the termination of blood transfusions, and the withholding of antibiotic treatment, amounts to euthanasia.

## Data Availability

The data and material are available upon reasonable request from the corresponding author. E-mail: andrzej.guzowski@umb.edu.pl.
